# Characterization of Plasma Extrachromosomal Circular DNA in Gouty Arthritis

**DOI:** 10.3389/fgene.2022.859513

**Published:** 2022-04-06

**Authors:** Jingyuan Pang, Xiaoguang Pan, Ling Lin, Lei Li, Shuai Yuan, Peng Han, Xiaopeng Ji, Hailong Li, Can Wang, Zhaobin Chu, Haoru Wu, Guangyi Fan, Xiao Du, Aichang Ji

**Affiliations:** ^1^ Medical School, Shandong University of Traditional Chinese Medicine, Jinan, China; ^2^ Lars Bolund Institute of Regenerative Medicine, Qingdao-Europe Advanced Institute for Life Sciences, BGI-Qingdao, Qingdao, China; ^3^ BGI-Qingdao, BGI-Shenzhen, Qingdao, China; ^4^ College of Life Sciences, University of Chinese Academy of Sciences, Beijing, China; ^5^ Emergency Department, Qingdao Third People's Hospital, Qingdao, China; ^6^ Shandong Provincial Key Laboratory of Metabolic Diseases and Qingdao Key Laboratory of Gout, The Affiliated Hospital of Qingdao University, Qingdao, China; ^7^ Institute of Metabolic Diseases, Qingdao University, Qingdao, China; ^8^ State Key Laboratory of Medicinal Chemical Biology and College of Pharmacy, Nankai University, Tianjin, China; ^9^ BGI-Shenzhen, Shenzhen, China

**Keywords:** eccDNA, gouty arthritis, inflammation, plasma DNA topologics, EccDNA-associated genes

## Abstract

**Objective:** Extrachromosomal circular DNA elements (eccDNAs) are known for their broad existence in cells and plasma, which may potentially play important roles in many biological processes. Our aim was to identify potentially functional or marked eccDNAs in gout patients.

**Methods:** The Circle-Seq approach was applied for eccDNA detection from plasma in acute gout patients and healthy controls. Further analysis was performed on the distribution of genomic elements and eccDNA gene annotations in two groups.

**Results:** We detected 57,216 and 109,683 eccDNAs from the acute gout and healthy control plasma, respectively. EccDNAs were mapped to the reference genome to identify diverse classes of genomic elements and there was no significant difference of eccDNAs on genomic element annotation between gout and control group. A total of 256 eccDNA-associated genes were detected as gout unique eccDNA genes, including COL1A1 and EPB42, which potentially contribute to hyperuricemia and gout, and a couple of genes involved in inflammation or immune response. Enrichment analysis showed that these eccDNA genes were highly correlated with defense response, stress response, and immune and inflammatory responses, including T cell receptor signaling pathway, Fc epsilon RI signaling pathway, and JAK-STAT signaling pathway.

**Conclusion:** Our discovery reveals the novel potential biological roles of plasma eccDNAs in gouty arthritis.

## Introduction

Nowadays gout is the most common inflammatory arthritis in males, which is mainly triggered by the deposition of monosodium urate (MSU) crystals in tissues and joints (So and Martinon, 2017). The epidemiology worldwide shows an increase in the incidence and prevalence of gout worldwide (Kuo et al., 2015; Dalbeth et al., 2019). Current studies suggest that innate immunostimulatory activity, especially the MSU crystals-mediated NLRP3 inflammasome activation plays a vital role in gouty arthritis development. Many inflammatory cytokines, such as interleukin-1β (IL-1β) and IL-6, participate in this immune response, ultimately leading to gouty attacks (Dalbeth et al., 2016; Wu et al., 2020).

Genetic variation has an important impact on the risk of gout (Barnett, 2018; Major et al., 2018). In the past decades, several research *via* genome-wide association studies on gout have been reported, revealing some genetic variants or genes related to gout and serum urate levels (Major et al., 2018; Tin et al., 2019). However, their explanation of the pathogenesis of gout is still limited. Therefore, other pathogenic mechanisms of gout need to be explored urgently.

Extrachromosomal circular DNA elements (eccDNAs) are derived from genomic DNAs and range in size from a few hundred bases to megabases (Wang et al., 2021). EccDNAs are known for their broad existence across different species. There are increasing evidence showing that they play important functions in diverse biological processes, such as DNA damage repair (Dillon et al., 2015), hypertranscription (Dillon et al., 2015; Hull et al., 2019), homologous recombination (Gresham et al., 2010), replication stress (Dillon et al., 2015), gene amplification in cancer (Paulsen et al., 2018; Morton et al., 2019), and ageing (Zhu et al., 2017; Hull et al., 2019; Qiu et al., 2021). Recently, Zhang’s research team from Boston reported that eccDNA was potent innate immunostimulants (Wang et al., 2021). Their studies showed that eccDNAs could activate IFNα, IFNβ, IL-6 or TNF in bone marrow-derived dendritic cells (BMDCs), and marrow-derived macrophages (BMDMs) (Wang et al., 2021).

Here, we sequenced and investigated the eccDNAs in plasma from patients with acute gout and healthy humans. This is very different from previous studies of gout genetics that focused on chromosomal DNA. We detected a large number of diverse eccDNAs with two distinctive peaks at 201 bp and 339 bp. We found that eccDNAs were common in human plasma and distributed in all genomic structures, including genes, intergenic and repetitive regions. We identified 256 eccDNA-associated genes solely represented in gout patients compared to healthy controls, from which we identified a couple of genes involved in inflammatory response, including TLR6, IL2RA, PTGS1, MAPK13, CHRNA4, GCH1, and IL5. Our discovery reveals the novel potential biological roles of plasma eccDNAs in gouty arthritis.

## Methods

### Plasma DNA Extraction, eccDNA Purification, and Amplification

Fresh blood was collected from four acute gout patients and four healthy people. Plasma was acquired by centrifuging the fresh blood at 3000 rpm for 10 min. Cell free DNA (cfDNA) was extracted from 300 μL plasma using a MGIEasy Circulating DNA Extraction Kit (MGI-BGI, China). DNA content was measured by Qubit 3.0. The cfDNA was digested using Plasmid-SafeTM ATP-dependent DNase (Epicentre, E310K) at 37°C for 1.5 h with a final concentration of 0.4 U/µl to remove linear dsDNA. After inactivating the ATP-dependent DNase at 70°C for 30 min, the remaining cfDNA was amplified using the REPLI-g Single Cell Kit (Qiagen, Cat #150343) at 30°C for 8 h. The amplified products were purified using Agencourt AMPure XP (Beckman Coulter, A63881) and quantified using Qubit 3.0.

### Library Preparation and Sequencing

The obtained eccDNA-enriched DNAs were fragmented into 100 to 300 bp using sonication (Covaris, Inc. Woburn, MA). We took 30 ng of the fragmented DNA to prepare DNA library using the MGIEasy DNA Library Preparation Kit. The library was sequenced using the MGISEQ-2000.

### Preprocess of Sequencing Data

The quality control of sequencing reads was performed by Fastqc-v0.11.3. Fastp-0.19.6 was used for filtering and removing low quality reads with default parameters. In order to identify circular DNA from Circle-seq data, we aligned all reads to the two-plasmid sequences (*P895 and P1035*) and the whole human genome sequence (GRCh38 download from UCSC) using BWA-MEM (v0.7.17) (Li, 2013) with default parameters, separately. To comply with the requirements of the Circle-Map (Prada-Luengo et al., 2019) pipeline (v1.1.4), we added the parameter -q to the BWA-MEM when reads were aligned to the human genome. We got two BAM files via sorting by reads names and coordinates.

### Circular DNA Identification

We used the Circle-Map pipeline for eccDNA detection (Prada-Luengo et al., 2019). All the details were the same as the description from GitHub-wiki (https://github.com/iprada/Circle-Map/wiki/Tutorial:-Identification-of-circular-DNA-using-Circle-Map-Realign). Filtering was conducted to improve the accuracy with parameters as followed: 1) split reads >= 2, 2) Circle score >=200, 3) Coverage increments in start coordinate >= 0.33, 4) Coverage increments in end coordinate >= 0.33, 5) Coverage continuity <= 0.1, and 6) Standard deviation <Mean coverage. All eccDNA regions that passed the filtering conditions were used for downstream analysis.

### Analysis of eccDNA

Using the Ensembl database assembly GRCh38, we identified gene positions on chromosomes. Genes were annotated using the UCSC table-browser (https://genome.ucsc.edu/cgi-bin/hgTables/). Gene ontology (GO) and Kyoto Encyclopedia of Genes and Genomes (KEGG) pathway enrichment analyses were performed based on the differentially expressed eccDNA-associated genes using Gene Set Enrichment Analysis (GSEA, http://www.gsea-msigdb.org/gsea/msigdb/annotate.jsp). The genes differed between groups were plotted using R package ComplexHeatmap (v2.11.1). GSEA annotation results were plotted using R package ggplot2 (v3.3.5).

### Motif Signature

We got the 15 bp upstream and downstream sequences flanking the junction of all merged eccDNAs using Circle-Map with bedtools-flank/slop/getfasta (v2.25.0) (Quinlan and Hall, 2010) functions. The start and end sequence motifs in R were generated using the ggseqlogo version 0.1 package (Wagih, 2017).

### Statistical Analysis

Statistical analyses were conducted using R version ≥4.1.1. The difference between two groups was checked using Wilcoxon rank-sum test. Correlation analysis were performed by Pearson correlation test. *p* value <0.05 was considered statistically significant.

## Results

### Identification and Distribution of eccDNA in Plasma

**FIGURE 1:**
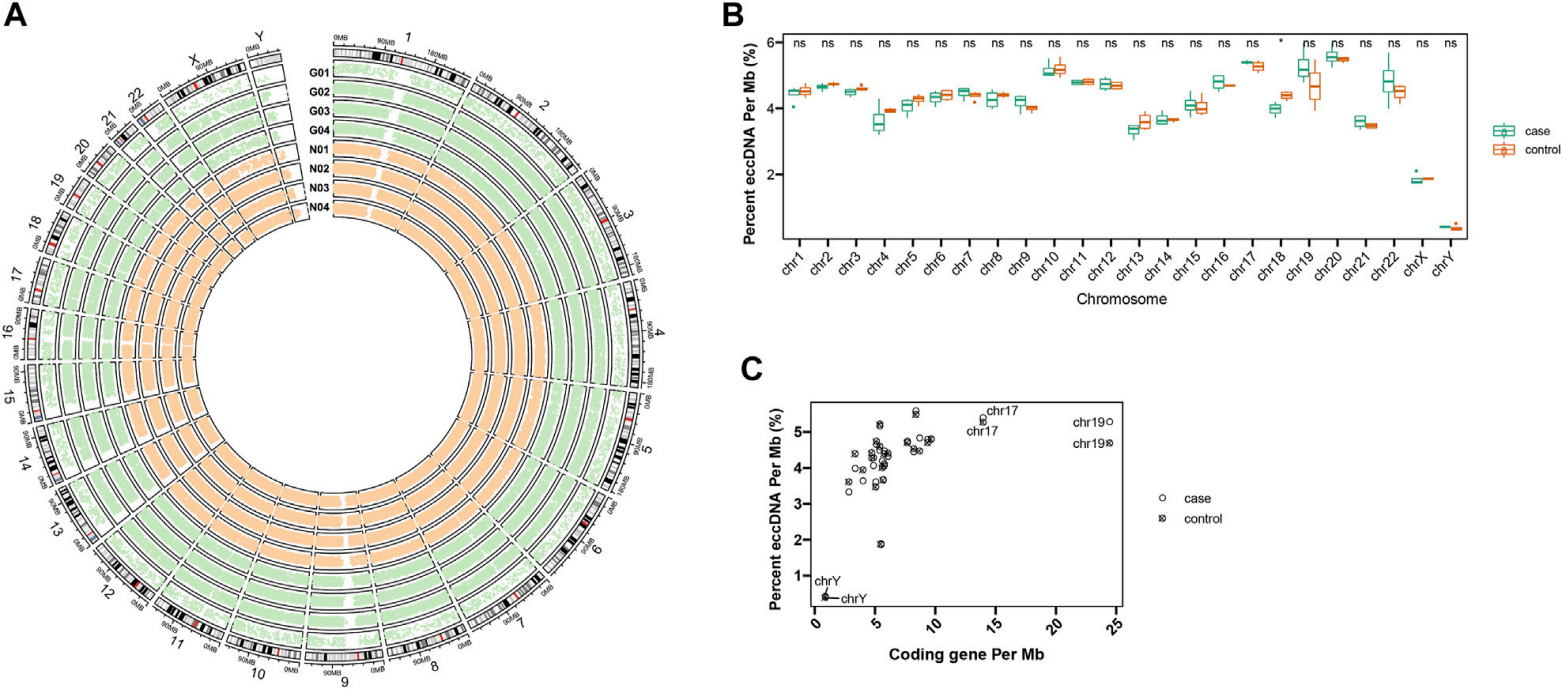
Distribution of plasma eccDNAs across human chromosomes. **(A)** The circos plot of eccDNA distributions from four acute gout patients (G01-G04) and four healthy controls (N01-N04). **(B)** The eccDNA distributions of gout and control on each chromosome. Ns stands for there are no significant differences of eccDNA distribution between control and gout group. * shows there are significant differences of eccDNA distribution between control and gout group on chromosome 18. **(C)** The frequency of eccDNAs per Mb and coding genes per Mb on each chromosome.

Circle-Seq method was applied for eccDNA detection from plasma from one group of acute gout (*n* = 4) and another group of healthy people (*n* = 4). Acute gout presents as an acutely painful joint inflammation. A total of 166,899 eccDNAs were identified from these eight plasma samples, showing eccDNAs were common in human plasma. We detected 57,216 and 109,683 eccDNAs from acute gout and healthy control plasma, respectively. Plasma eccDNA sequences from gout and healthy controls were distributed across all chromosomes with a combined length of 5.73 and 10.84 Mb of the human genome, respectively (Figure 1A). When checking frequency of eccDNAs per Mb on each chromosome, chromosomes 20, 17, 10, and 19 displayed a relatively higher eccDNA frequency compared to the others (Figure 1B). Across the chromosomes, chromosome 17 and 19 showed high coding genes per Mb along with the highest eccDNA frequency (Figures 1B,C). This pattern was in line with previous observation in human muscle and blood eccDNA study (Møller et al., 2018), suggesting that features of coding genes might play a role on eccDNA formation. Interestingly, we found that chromosome 18 were differentially represented in eccDNA frequency between gout and control groups, while no significant differences were detected on other chromosomes. (Figure 1B; chromosome 18: *p* < 0.05).

### Genomic Characteristics of Plasma eccDNA

**FIGURE 2:**
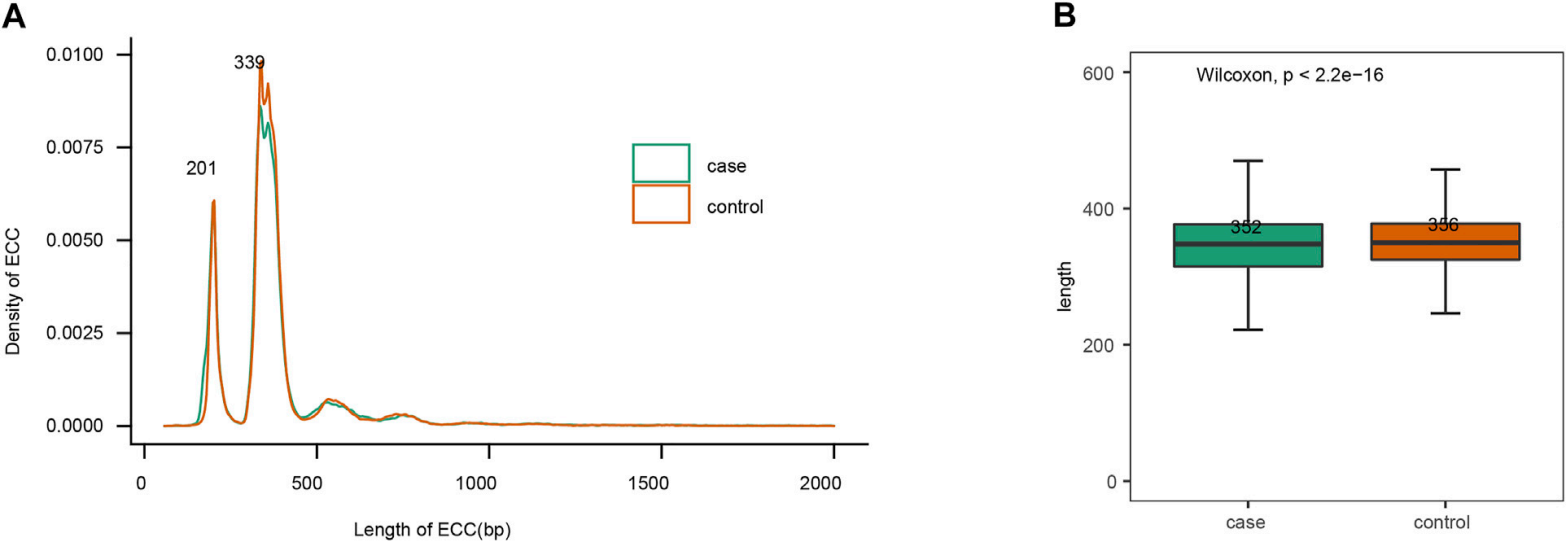
EccDNAs size features. **(A)** Size distribution of plasma eccDNAs < 2 kb. **(B)** Length comparison of eccDNAs < 2 kb between gout and control group.

Size distribution of eccDNAs from these eight plasma samples showed comparable size distributions between the gout and healthy control group (Figure 2A). The plasma eccDNAs displayed two distinctive peaks at 201 bp and 339 bp, and the 339 bp peak was more predominant than the 201 bp peak (Figure 2A). This size signature was in concordance with previous observations studying plasma eccDNA (Kumar et al., 2017; Sin et al., 2020). When comparing the eccDNA size <2 kb between the gout and control group, we detected significantly longer eccDNA sequences in the healthy control by Wilcoxon rank sum test (*p*-value < 2.2e -16; Figure 2B).

#### Genomic Annotation of eccDNA

**FIGURE 3:**
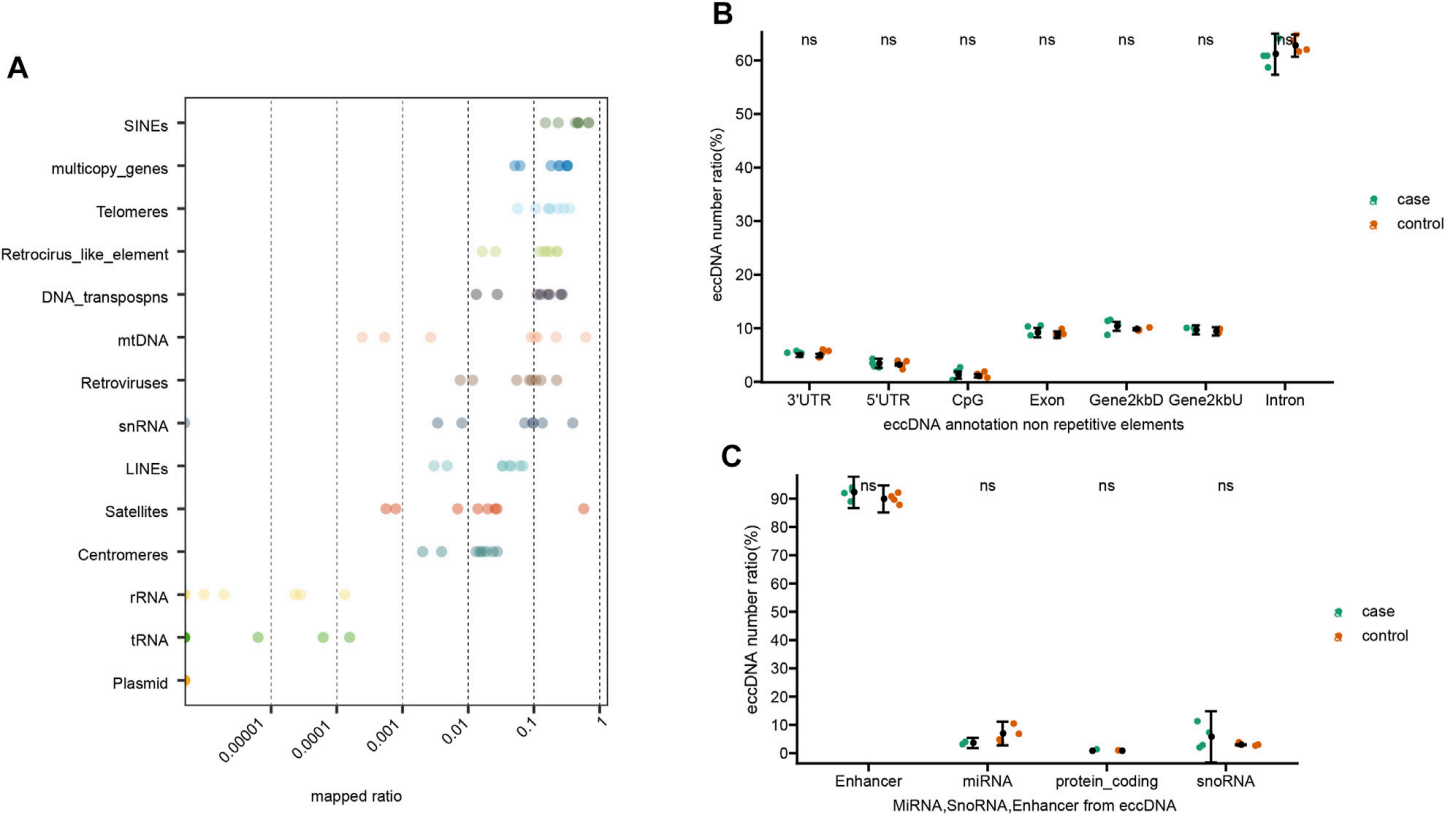
Genomic elements of annotated eccDNAs. **(A)** EccDNA annotation on repetitive elements. **(B)** EccDNA annotation on non-repetitive elements. **(C)** MiRNA, SnoRNA, Enhancer from eccDNA. Ns stands for there are no significant differences of eccDNA distribution between control and gout group.

The overall detected eccDNAs were mapped to the reference genome to identify diverse classes of genomic elements (Figure 3). Firstly, a substantial number of eccDNA sequences were mapped to diverse repetitive regions. The plasma eccDNA molecules were mapped to SINEs (0.45%), telomeres (0.19%), Retroviruses (0.09%), LINEs (0.04%), satellites (0.08%), and centromeres (0.02%; Figure 3A), detected from all types of repetitive regions in human genome. This was in concordance with the knowledge that a large fraction of eccDNAs derive from repetitive sequences (Ling et al., 2021). Apart from that, 70.23% of all eccDNAs were mapped to genic regions. Among that, 9 and 62% were represented in introns and exons, respectively. Five percent and 3% were mapped to 3′UTRs and 5′UTRs, respectively (Figure 3B). Some plasma eccDNAs were also detected from enhancers (Figure 3C). No significant differences were detected between gout and control group in genomic element annotation (Figures 3B,C).

### Trinucleotide Motifs Flanking eccDNA Junctions

**FIGURE 4:**
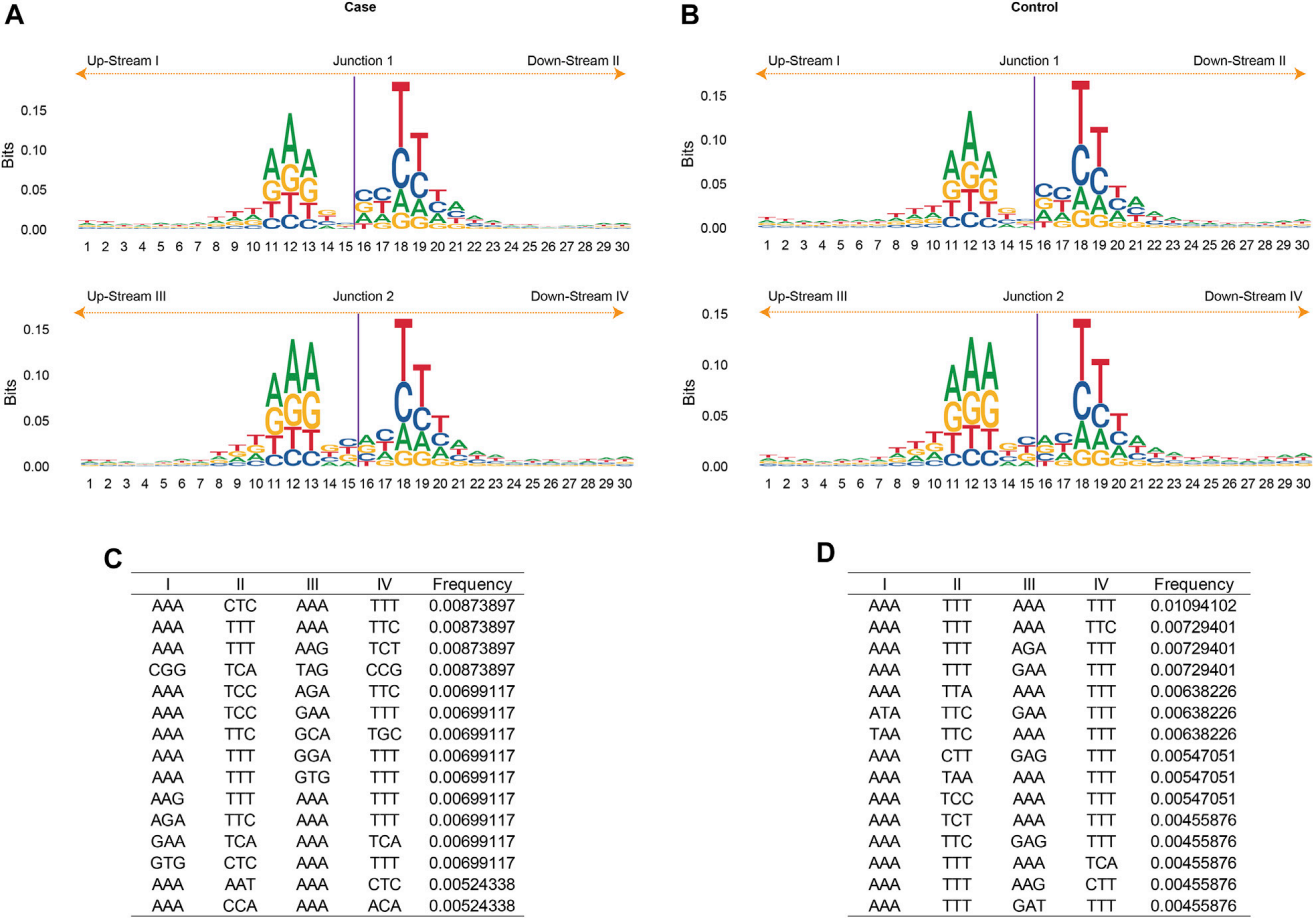
The motif analysis of eccDNA junctions. The nucleotide composition from 15 bp upstream to 15 bp downstream of the start (Junction 1) and end position (Junction 2) of the eccDNA junction in acute gout **(A)** and healthy control **(B)**. The frequency of base distribution at each position is plotted as a stacked histogram. The size of four bases at each position in the figure reflects their frequency. The 3-bp motifs flanking the start and end site in acute gout **(C)** and healthy control **(D)**. Data were ranked by motif frequencies. I, II, III, IV refers to 3-bp upstream of start site, 3-bp downstream of start site, 3-bp upstream of end site, and 3-bp downstream of end site, respectively.

The eccDNA junction was defined as the site where two ends of a genomic sequence ligated to generate an eccDNA. Therefore, the sequence pattern flanking the eccDNA junctional sites was important as it may shed light on exploring the mechanisms of eccDNA biogenesis. The nucleotide composition from 15 bp upstream to 15 bp downstream of the start and end position of the junction of all merged eccDNAs was analyzed and plotted in Figures 4A,B. The frequency of base distribution at each position flanking the start and end site is plotted as a stacked histogram. The relative size of four bases at each position in the figure indicates their frequency. The height of each letter is directly proportional to the frequency of the corresponding base at that position. The bases in each position are arranged from large to small, which can identify the conservative sequence from the bases at the top. As shown in Figures 4A,B, both the start and end positions of eccDNA junctions were flanked by a pair of high-frequency trinucleotide segments with 4-bp “spacers” in between. These trinucleotide segments, which were located at 11–13 bp and 18–20 bp from junction 1 and junction 2 (Figure 4A), were termed as motifs I, II, III, and IV in Figure 4C. The sequences of these four trinucleotide segments with the top 20 frequencies were listed in Figures 4C,D for gout and control independently. A recurrent pattern was observed from these sequences: sequence AAA showed high frequency in motifs I and III, whereas sequences TTT and TTC showed dominance in motifs II and IV.

### Functional Analysis of eccDNA

**FIGURE 5:**
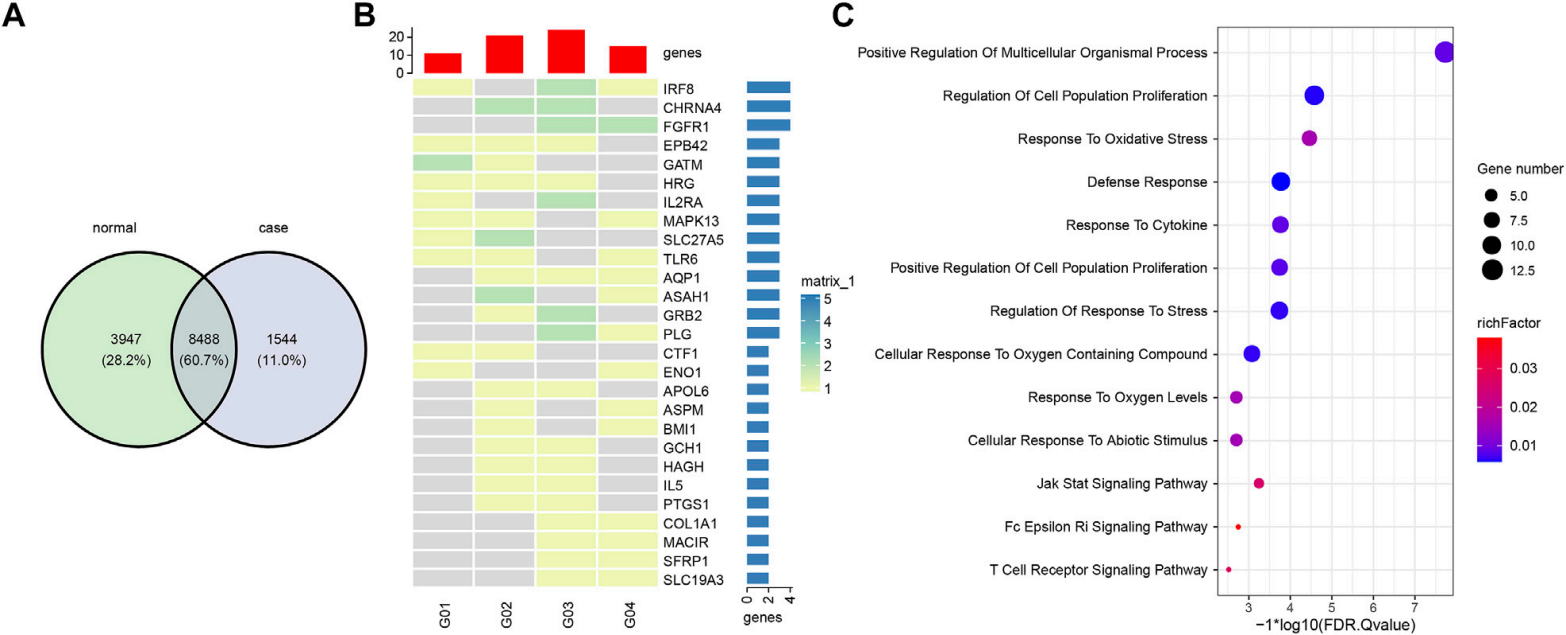
Comparison and functional analysis of eccDNA genes. **(A)** Shared and unique eccDNA genes between gout and healthy controls. **(B)** A heatmap of 27 gout unique eccDNA genes involved in inflammatory response or potentially gout-related diseases. **(C)** Functional enrichment of 27 gout unique eccDNA genes.

We used the overall 166,899 eccDNAs for gene annotation and annotated 13,979 genes in total. Among that, 8,488 (60.7%) genes were shared by all eight samples. 1,544 (11%) and 3,947 (28.2%) genes were only represented in eccDNAs from gout and healthy control plasma, respectively (Figure 5A). To investigate the differentially represented eccDNA genes in gout patients, eccDNA genes that were represented in at least two gout patients but absent from the healthy control were defined as gout unique eccDNA genes. We ended up with 256 eccDNA genes only represented in gout, from which 27 genes were identified involved in inflammatory response or potentially gout-related diseases (Figures 5B,C). Specifically, Gene TLR6, IL2RA, PTGS1, MAPK13, CHRNA4, GCH1, and IL5 play a role in inflammation or immune response. COL1A1 and EPB42, which potentially contribute to hyperuricemia and gout, were also revealed by gout eccDNAs. A list of diverse genes associated with rheumatoid arthritis were detected, including PTGS1, MACIR, APOL6, IRF8, AQP1, ASAH1, ENO1, FGFR1, GRB2, HAGH, HRG, MAPK13, PLG, and SFRP1. Other detected genes including SLC19A3, CTF1, and GATM are related to kidney diseases. GO and KEGG enrichment analysis showed that these 27 gout-unique eccDNA genes were highly correlated with defense response, stress response, and immune and inflammatory responses, including T cell receptor signaling pathway, Fc epsilon RI signaling pathway, and JAK-STAT signaling pathway (Figure 5C).

## Discussion

The eccDNA is reported to be ubiquitous in eukaryotes (Paulsen et al., 2018; Wang et al., 2021). It is not only present in tumor tissues, but also abundant in plasma and serum of normal tissues (Zhu et al., 2017). The eccDNA can cause gene overexpression due to its high copy number, enhanced transcriptional activity, and enhanced activity of chromosomal and extrachromosomal genes (Nathanson et al., 2014; Zhu et al., 2017; Paulsen et al., 2018; Zhu et al., 2021). The high occurrence of cell-free eccDNAs in the blood system suggest eccDNAs may play vital roles in biological processes, which may potentially act as novel biomarkers for early disease detection, risk assessment, and monitoring of drug treatment response (Paulsen et al., 2018).

The occurrence of gout is presented by the trigger of the inflammatory response, which is induced by the deposition of urate and the overactivation of the innate immunity sequentially (Barnett, 2018; Dalbeth et al., 2019; Dalbeth et al., 2021). A series of signaling pathways are involved in this process. Acute gout attacks depend on the activation of macrophage TLRs/NF-κB signaling pathway (signal 1) and NLRP3 inflammasome complex/IL-1β (signal 2) dual signaling pathways (Wu et al., 2020; Clavijo-Cornejo et al., 2021). As MSU crystals stimulate TLR2 and TLR4 in macrophages, signal 1 is activated, generating the components ASC, Pro-Caspase 1, NLRP3, and Pro-IL-1β. Once MSU crystals enter macrophages by endocytosis, the NLRP3 inflammatory complex is activated. A series of signaling pathways involving proinflammatory cytokines and chemokines are initiated by the mature IL-1β, with neutrophils delivered to the crystal deposition site (So and Martinon, 2017; Dalbeth et al., 2021).

In this study, we report for the first time of the eccDNA characteristics in the acute phase of gout in blood. A couple of eccDNAs of inflammatory factors or genes were different in gout patients and healthy controls. The TLR6, IL2RA, PTGS1, MAPK13, and IL5, which were important in inflammatory response, were only discovered in the acute gout patients in our study. In particular, TLR6 is involved in the key signaling pathway of gout inflammatory response, the NF-kappaB signaling pathway (Hou et al., 2008; Dalbeth et al., 2019). The contents of interleukin 2 (IL-2) were significantly higher than the blank group in acute gouty arthritis rats (Sun et al., 2015). IL2RA is the receptor of IL-2, and IL-2 is the inflammatory markers of gout (Alzahrani et al., 2019). Studies also showed that Th17 was involved in gouty arthritis. The inflammatory Th17 was elevated in MSU crystal-induced arthritis in acute gout rats, which was in concordance with the development of inflammation in gouty arthritis (Dai et al., 2018). The IL5 and MAPK13 discovered in our study are involved in IL-17 signaling pathway (Hashimoto et al., 2005; Yue et al., 2021).

The eccDNA has been reported to activate IFNα, IFNβ, IL-6 or TNF as the potent innate immunostimulants dependent on eccDNA circularity (Wang et al., 2021). In our study, we discovered some eccDNAs associated with inflammatory genes in acute gouty arthritis patients, which might potentially play a role in the activation of the inflammatory response caused by urate crystals and serve as biomarkers for gouty arthritis. Limited by our sample size, this requires further experimental verification.

## Data Availability

The datasets presented in this study can be found in online repositories. The names of the repository/repositories and accession number(s) can be found below: https://db.cngb.org/cnsa, CNP0002557.
